# Polycaprolactone nanofibres loaded with 20(*S*)-protopanaxadiol for *in vitro* and *in vivo* anti-tumour activity study

**DOI:** 10.1098/rsos.180137

**Published:** 2018-05-02

**Authors:** Dan-qing Liu, Zhi-qiang Cheng, Qing-jie Feng, He-jie Li, Shu-feng Ye, Bo Teng

**Affiliations:** 1 Department of Otolaryngology Head and Neck Surgery, The Second Hospital, Jilin University, Changchun 130041, People's Republic of China; 2 College of Resources and Environment, Jilin Agriculture University, Changchun 130118, People's Republic of China

**Keywords:** polycaprolactone nanofibres, 20(*S*)-protopanaxadiol, anti-tumour

## Abstract

In this work, 20(*S*)-protopanaxadiol (PPD)-loaded polycaprolactone (PCL) nanofibres were successfully fabricated by the electrospinning technique using Tween 80 as a solubilizer. Firstly, smooth and continuous nanofibres were collected using suitable solvents and appropriate spinning conditions. Secondly, nanofibre mats were characterized by scanning electron microscopy, thermogravimetric (TG) analysis, Fourier transform infrared spectroscopy and mechanical testing. Finally, nanofibrous membranes were evaluated using water contact angle, *in vitro* drug release, biodegradation test, *in vitro* and *in vivo* anti-tumour activity and cell apoptosis assay. Scanning electron microscopic observations indicated that the diameter of the drug-loaded nanofibres increased with the increase of drug concentration. TG analysis and mechanical test showed that nanofibres were equipped with great thermal and mechanical properties. Biodegradation test exhibited that the structure of fabricated nanofibres had a certain degree of change after 15 days. An *in vitro* release study showed that PPD from drug-loaded nanofibres could be released in a sustained and prolonged mode. The cytotoxic effect of drug-loaded nanofibre mats examined on human laryngeal carcinoma cells (Hep-2 cells) demonstrated that the prepared nanofibres had a remarkable anti-tumour effect. Meanwhile, the drug-loaded fibre mats showed a super anti-tumour effect in an *in vivo* anti-tumour study. All in all, PCL nanofibres could be a potential carrier of PPD for cancer treatment.

## 1. Introduction

Currently, cancer is still a worldwide issue that humankind has been confronted with. Human health and quality of life have been seriously threatened by cancers due to their intrinsic characteristics: growing fast, easy to transfer and relapse. According to reports, the incidence and mortality rates of cancers are still on the rise [1]. It is well known that the treatment of malignant tumour at present includes surgery, radiotherapy, chemotherapy, hyperthermia, immunotherapy, hormone therapy, stem cell therapy and combinations of these modalities [2]. Clinical workers choose treatment strategy according to the patient's specific situation, and the most common therapeutic method is chemotherapy in combination with surgery or radiation therapy [3]. Unfortunately, there still exist many problems for chemotherapy in spite of great efforts having been made. Now, under normal circumstances, a long-term, reduplicative and large dose of chemotherapy drugs are needed for cancer patients in order to achieve effective anti-tumour effect. However, these drugs produce severe toxicity in healthy tissues that should not be ignored. What is more, the systematic administration of large dose anti-neoplastic drugs may lead to death [4]. Therefore, appropriate drug carrier has attracted more and more attention in the biomedical field to overcome these problems.

Herbal materials have been used in medical field for a long time in East Asian countries. 20(*S*)-Protopanaxadiol (PPD), an active ginseng metabolite, is the final form of protopanaxadiol saponins metabolized by human intestinal microflora [5]. As far as we know, it exhibits tremendous anti-cancer effects against various tumour types [6–8]. However, the clinical use of PPD is limited by its poor water solubility (3 µg ml^−1^) and low oral bioavailability [9]. A large number of studies have been made to solve the above problem. Jin *et al*. described a nanoparticulate drug-delivery system for PPD to enhance its oral bioavailability [10]. Thus, we expect to prepare a drug carrier to control the release of PPD and this carrier can act directly on the target site to improve the utilization rate.

Electrospinning is an emerging technology which is versatile, inexpensive and straightforward that can fabricate ultrafine fibres with diameters ranging from several nanometres to a few micrometres and with steerable surface morphology [11]. These fibres are generated by electrostatic repulsion and the Coulomb force due to an external electric field applied to a polymer solution or melt. A polymer jet is created by applying a critical potential between a metallic needle and a grounded collector, and the fabricated nanofibres can be collected at the collecting device [12]. Electrospun nanofibres have been extensively used in many fields, such as energy-related applications [13], tissue engineering scaffolds [14,15], wound dressings [16], vascular grafts [17,18], drug delivery [19–21] and biosensor applications [22]. Electrospinning nanofibres as pharmaceutical carriers have obtained more and more attention in biomedical field in recent years. There are plenty of advantages for electrospun nanofibres as a delivery vehicle such as high loading capacity and encapsulation efficiency, high surface area, targeted release and ability to be modified for regulated drug delivery [23,24]. The large surface area of polymer nanofibre mats allows increased close interaction of therapeutic agents with tissues [25]. Besides, biocompatible and biodegradable polymer nanofibres with sizes less than 1 µm are especially useful in the field of medicine, because these nanomaterials replicate components of *in vivo* cellular and molecular environment [26]. Polycaprolactone (PCL) is a semi-crystalline, environment-friendly, biodegradable and biodegradable compatible polymer which has been approved by the Food and Drug Administration in the USA for biomedical uses [27]. In addition to the above characteristics, PCL can be easily electrospun and has the ability to generate long-term sustained drug delivery [28]. Tween 80 is a polyethylene sorbitol ester which is widely used in biochemical fields including for solubilizing proteins, isolating nuclei from cells in cell culture, growing tubercule bacilli, and emulsifying and dispersing substances in medicinal and food products [29]. Therefore, PPD-loaded PCL nanofibres have the ability to be used in cancer treatment when Tween 80 is used as a solubilizer.

In this study, for the first time, PPD-loaded PCL (PCL/PPD) electrospun nanofibres were successfully fabricated, and the anti-cancer effect of Hep-2 cells was evaluated. Morphology, physical and drug release properties, and water contact angle were also characterized. The results demonstrated that these drug-loaded nanofibres had a sustained release behaviour of PPD. Further *in vitro* anti-tumour activity and cell apoptosis assay indicated that PCL/PPD nanofibres display a markedly therapeutic effect for tumour cells. Meanwhile, drug-loaded nanofibrous membranes showed an effective anti-tumour effect in *in vivo* experiment, implying that they could be a potential carrier of PPD for cancer treatment.

## 2. Material and methods

### 2.1. Materials

PCL (average molecular weight Mw = 8000) and 3-(4,5-dimethyl-2-thiazolyl)-2,5-diphenyl-2-H-tetrazolium bromide (MTT) were purchased from Sigma-Aldrich. Hexafluoroisopropanol (HFIP, 99.5%) was obtained from Aladdin. Dulbecco's modified Eagle's medium (DMEM) and fetal bovine serum (FBS) were purchased from HyClone. Trypsin and phosphate-buffered saline (PBS) buffer (pH 7.4) were bought from Solarbio. PPD (greater than 98%) was provided from the College of Chemistry, Jilin University. All the chemical reagents were used as received without further purification. Female BALB/c nude mice, weighing 20 ± 2 g, were purchased from Vital River Laboratories (Beijing, China). All of the experiments were performed in accordance with the guide for the care and use of laboratory animals promulgated by the National Science and Technology Commission of China, 1988 as Decree no. 2.

### 2.2. Preparation of nanofibres

PCL nanofibres and PCL/PPD nanofibres were prepared by the electrospinning technique. PCL was dissolved in HFIP, and the concentration of solution was 3% (w/w). Then, Tween 80 (0.1% based on the content of PCL) and different concentrations of PPD (i.e. 3%, 5%, 10% and 20% based on the solid content in the polymer solution) were added into the solution. The solution was stirred gently at room temperature for 12 h and was placed at room temperature for another 12 h before electrospinning. After the preliminary experiment, optimal spinning conditions were found: 20 kV (applied voltage), 20 cm (distance between nozzle and collector), 22°C (spinning temperature), 18% (spinning humidity), 21 G needle and the flow rate varied between 0.8 and 1 ml h^−1^.

### 2.3. Characterization

The morphological investigations of nanofibres were carried out by a scanning electron microscope (SEM; Shimadzu X-550). At least 30 nanofibres were chosen from different SEM images, and the diameter of nanofibre was measured using Nano Measurer software. Mechanical properties of electrospun membranes were measured by a tensile tester (WDW-X). At least three samples of individual membranes were tested. The water contact angle of nanofibres was evaluated by a contact angle analyser (Kino SL200B, USA). Thermogravimetric analyser (TG-DTA, HCT-3) was applied to evaluate the thermal stability of nanofibre mats. The infrared spectra of nanofibres were acquired by using a Fourier transform infrared spectrometer (FTIR-650). To accomplish the biodegradability test, fibres was immersed in PBS (pH 7.4) and cultivated for a period of 15 days at 37°C. Then, the nanofibre mats were removed from PBS and dried in vacuum for 24 h before fibre morphology was observed by SEM.

### 2.4. Drug release analysis

The PPD-containing membranes were accurately weighed (every sample contained the same amount of PPD). And then, the nanofibres were immersed in a release medium (0.5% Tween 80 in PBS, pH 7.4) and gently stirred at 37°C with a speed of 100 rpm. At a certain time interval, a certain amount of solution was removed and replaced with the same volume of a fresh medium. A high-performance liquid chromatography system (Waters e2695) was used to examine the drug release profiles of PCL/PPD nanofibres. The separation of PPD was performed with (Hypersil BDS) C18 column (5 µm, 4 mm × 250 mm) and it was spotted at 203 nm wavelength.

### 2.5. Cell culture

Laryngeal carcinoma cell line Hep-2 was obtained from Cell Lines Bank, Shanghai Institute of Biological Sciences. Cells were cultured in DMEM supplemented with 10% FBS and antibiotics (100 IU ml^−1^ of penicillin and 100 mg ml^−1^ of streptomycin). The cells were cultured at 37°C in a humidified incubator containing 5% CO_2_.

### 2.6. *In vitro* anti-tumour activity, cell apoptosis assay and cell morphology

MTT assay was applied to explore the viability of the Hep-2 cells managed with different samples. Cells were harvested using trypsin solution and then resuspended in fresh DMEM. The samples were sterilized under UV light for 24 h before cell seeding. In brief, Hep-2 cells at a density of 1 × 10^4^ cells well^−1^ were seeded into 96-well plates and cultivated at 37°C overnight. Then, cells were exposed to free PPD (positive control), different concentrations of drug-loaded fibres, PCL nanofibre mats and pure medium (negative control). The total concentration of PPD was 20 µg ml^−1^, 30 µg ml^−1^ and 40 µg ml^−1^, respectively. After the cells were cultured for 24 h, 72 h and 120 h, nanofibres were removed carefully and then 20 µl of MTT (0.5 mg ml^−1^) was added to each well. The cells were kept to incubate for another 4 h. Thereafter, the medium was discarded and 150 µl of dimethylsulfoxide was added to dissolve the resultant blue formazan products by stirring for 10 min. The optical density value was measured using a microplate reader (Biorad) at 490 nm. The relative cell viability was expressed as a percentage of the control group, and the mean value was obtained from at least five parallel samples.

Fluorescence microscopy detects apoptosis by calcein-AM/propidium iodide double staining. Cells were seeded in 24-well culture plates at a density of 2 × 10^4^ cells per well and cultivated at 37°C overnight. Then, PCL/PPD nanofibres were added to the cells, and samples were incubated for 72 h. Thereafter, cells were stained using a LIVE/DEAD Viability/Cytotoxicity Assay Kit (KGAF001). Those cells were observed under fluorescence microscopy (Nikon Eclipse Ti-s).

To confirm the anti-tumour effect of each sample, the cell apoptosis assay was assessed with flow cytometry. Hep-2 cells were seeded in six-well culture plates at a density of 2 × 10^5^ cells per well and cultivated at 37°C overnight. PCL/PPD nanofibre mats were added at an equivalent PPD dosage of 20 µg ml^−1^ to the cells. After 72 h, the rate of apoptosis was detected using an Annexin V-FITC-PI Apoptosis Detection Kit (GenStar) in compliance with the product description. And then, it was analysed by flow cytometry (Becton Dickinson, San Jose, CA, USA).

To observe the morphological change of the cells under treatment with PPD in an intuitive way, Hep-2 cells were seeded in six-well culture plates at a density of 1 × 10^5^ cells per well. After cell attachment, the cell morphology was examined under a microscope. Then, cells were exposed to a pure medium and PCL/PPD nanofibres with a PPD concentration of 40 µg ml^−1^. After 72 and 120 h, nanofibres were removed carefully, and the morphology of the cells was examined under a microscope.

### 2.7. *In vivo* anti-tumour effect study

All of the BALB/c nude mice were subcutaneously inoculated with Hep-2 cells (1 × 10^7^ cells ml^−1^ medium, 100 µl per mouse) in the right axillae on day 0. Approximately 15 days after inoculation, xenografts in the BALB/c nude mice reached a volume of approximately 60 mm^3^ (labelled as day 0). Then, all the mice were randomized into four groups (*n* = 5) and numbered: control group, pure PPD group, PCL/PPD nanofibre group and PCL nanofibre group. After the preparation was completed, PBS was injected in the mice every 2 days for the control group. For the PPD group, the mice were injected with PPD (20 mg kg^−1^) every 2 days for three times. Meanwhile, the PCL/PPD and PCL nanofibre groups were implanted subcutaneously near the tumours. The PPD group and the PCL/PPD nanofibre group contained the same amount of PPD (20 mg kg^−1^). The size of tumours in all mice was measured every 3 days. At day 9, the mice were sacrificed. Then, the tumours were removed and weighed.

## 3. Results and discussion

### 3.1. Morphological properties of nanofibres

**Figure 1.**
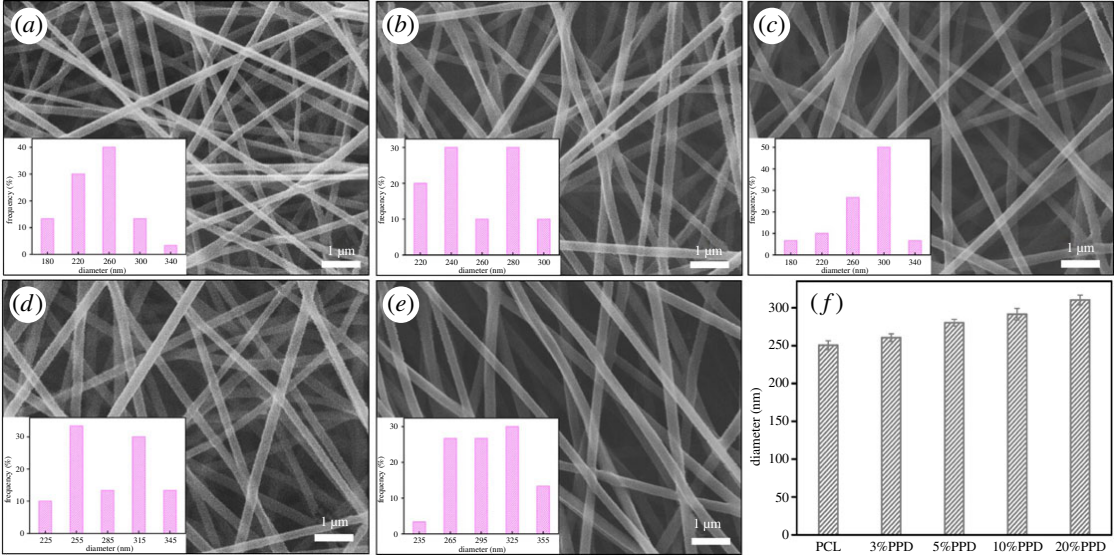
SEM images of nanofibres: (*a*) PCL, (*b*) PCL/3%PPD, (*c*) PCL/5%PPD, (*d*) PCL/10%PPD, (*e*) PCL/20%PPD. (*f*) Mean diameter of different nanofibres; the data are presented as average ± standard deviation (*n* = 3).

As is well known, the morphology of electrospun mats is influenced by various parameters. In this work, nanofibres were fabricated by electrospinning under the optimized parameters such as polymer concentration, voltage and distance. The obtained SEM images of nonafibres are shown in figure 1*a*–*e*. This revealed that all the nanofibres were randomly aligned, interconnected and continuous fibres. It can also be distinctly seen that smooth bead-free and relatively uniform fibrous diameter fibres were obtained. We can infer that the drug was successfully loaded on the nanofibre membrane from the SEM images. As shown in figure 1*f*, the incorporation of PPD into the PCL nanofibres increased their average diameter. As was expected, the addition of PPD increased the viscosity of polymeric solution. In the same spinning conditions, the diameter of the nanofibres increases with the polymer solution viscosity. As shown in figure 1*f*, the average diameter of PCL nanofibres and 3%, 5%, 10% and 20% drug-loaded samples were 250 ± 6 nm, 260 ± 5 nm, 280 ± 4.5 nm, 291 ± 7.6 nm and 310 ± 6.5 nm, respectively. In other words, through appropriate spinning parameters, smooth and uniform nanofibres were fabricated successfully for the subsequent experiments.

### 3.2. Biodegradation test of nanofibres

**Figure 2.**
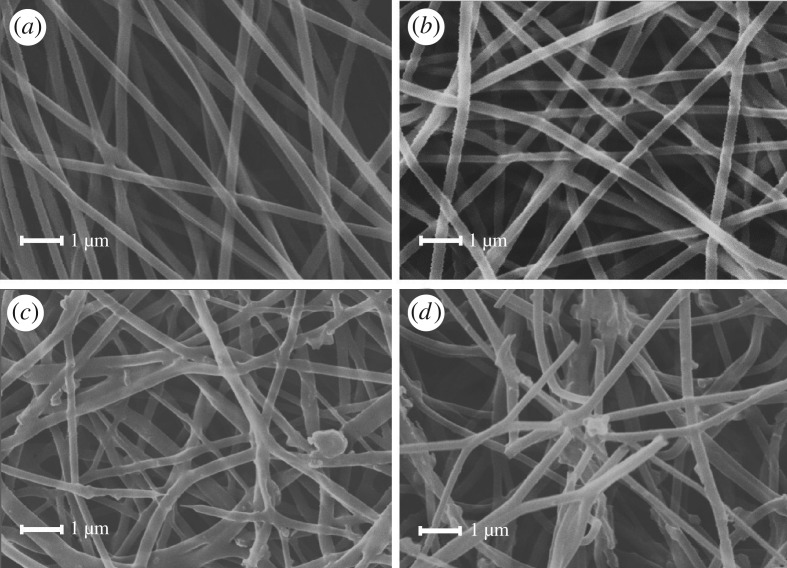
SEM images of PCL/20% PPD before and after immersion into PBS for several days. (*a*) PCL/20% PPD day 0, (*b*) PCL/20% PPD day 3, (*c*) PCL/20% PPD day 7 and (*d*) PCL/20% PPD day 15.

PCL has biodegradability besides good biocompatibility. And the biodegradable property is particularly important for a drug carrier. Nanofibre membranes produced by the preliminary experiment were soaked in 37°C PBS solution for 3 days, 7 days and 15 days. The structure and morphology of nanofibre membranes after treatment were studied by SEM. As shown in figure 2, the structure of drug-loaded fibres was changed and several nanofibres were broken down. Thus, it can be seen that these drug-loaded nanofibres could be biodegradable in the human body.

### 3.3. Hydrophilicity of the nanofibres

**Figure 3.**
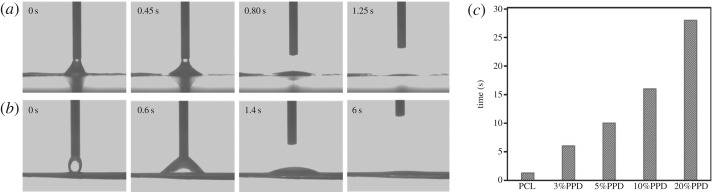
Contact angle measurement of nanofibres. (*a*) PCL nanofibres, (*b*) PCL/3% PPD and (*c*) comparison of different samples.

The wettability of the nanofibres is one of the most significant features for drug delivery. PCL, which is widely used in the biomedicine field for a long time, is a semi-crystalline lyophobic polymer. To improve the hydrophilicity, Tween 80 was added into the blend solution for electrospinning. We made liquid droplet contact with samples and measured their contact angle. Figure 3 exhibits the water contact angle of the nanofibre mat. And as shown in figure 3*c*, the time needed for liquid drops to be completely absorbed was 1.25, 6, 10, 16 and 28 s, which was prolonged with the increase of drug concentration. Unsurprisingly, the addition of PPD extends the period of the droplets absorbed by nanofibres, but it did not affect the final hydrophilicity of nanofibres.

### 3.4. Fourier transform infrared spectrometric analysis

**Figure 4.**
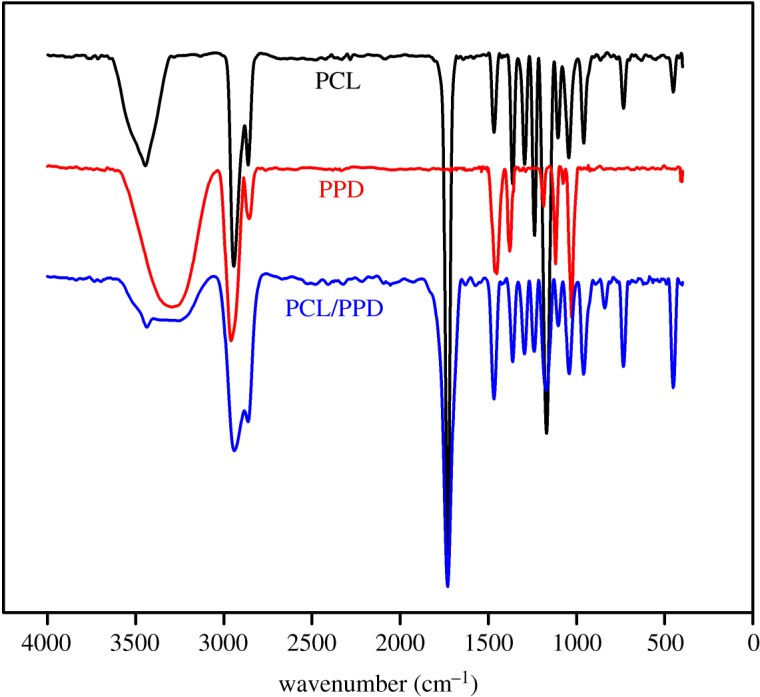
FTIR spectra of PCL nanofibres, free PPD and PCL/PPD nanofibres.

The FTIR spectra of samples are depicted in figure 4. The spectra of PCL/PPD nanofibres contain both the characteristic peaks of PPD and PCL. In the PCL nanofibre spectrum, asymmetric CH_2_ stretching appears at 2949 cm^−1^, symmetric CH_2_ stretching appears at 2865 cm^−1^ and carbonyl stretching appears at 1727 cm^−1^ [30]. A broad absorption band at 3400–3650 cm^−1^ is due to the terminal hydroxyl group [31]. The band at 1298 cm^−1^ was regarded as the crystallinity change in PCL in previous literature [32]. The results show that PCL was successfully combined. In the spectrum of PPD, the peak at 3288 cm^−1^ is attributed to the –O–H stretching. All of the above typical peaks were detected in the spectrum of the PCL/PPD nanofibres. Thus, it can be proved that all the experiment materials were successfully blended together.

### 3.5. Thermogravimetric analysis

**Figure 5.**
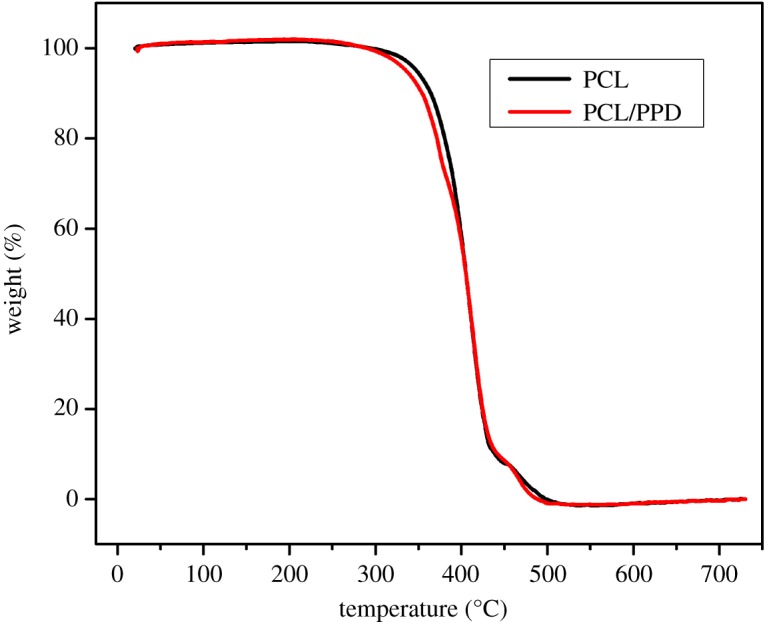
Thermogravimetric analysis thermograms of PCL and PCL /PPD nanofibres.

Thermogravimetric analyses of PCL and PCL/PPD nanofibres were performed to probe their thermal properties. Figure 5 shows that the degradation of both samples occurred between 300 and 550°C, which were independent from water losses. The result reveals that the drug-loaded nanofibre has similar thermal stability to PCL nanofibre. It is possible to confirm that the addition of PPD to PCL nanofibres did not influence the thermal stability of the fibres.

### 3.6. Tensile strength of the nanofibres

**Figure 6.**
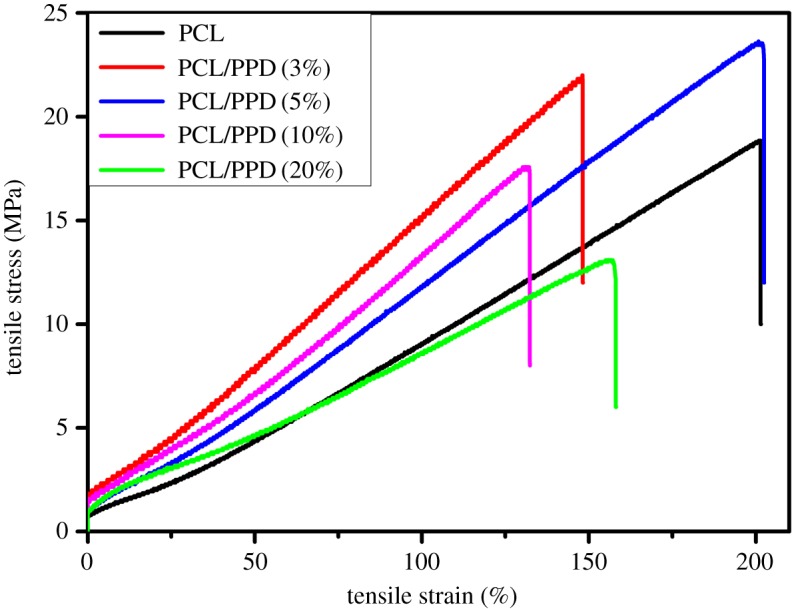
Typical stress–strain curves of the PPD-loaded PCL membranes and PCL membranes.

Tensile strength test was carried out to explore the mechanical characterization of nanofibres. Figure 6 shows the nonlinear stress–strain curves for PCL nanofibres and drug-loaded nanofibres with different concentrations. PCL nanofibres showed a tensile strength of 18.85 MPa and a tensile strain of 201%. Drug-loaded nanofibres (PPD concentration was 3%, 5%, 10% and 20%) showed tensile strength of 21.8 MPa, 23.5 MPa, 17.2 MPa and 13.1 MPa, respectively, and tensile strain of 148%, 202%, 132% and 158%, respectively. The results indicate that as the concentration of the drug increased, the trend of the tensile strength of the PPD-loaded nanofibre mats decreases after an increase compared with the PCL membrane. Thus, we can infer that in order to obtain drug-loaded nanofibres with great mechanical property, the concentration of PPD should not be too high.

### 3.7. *In vitro* release of 20(*S*)-protopanaxadiol

**Figure 7.**
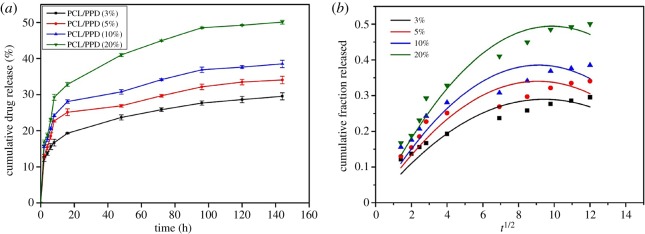
(*a*) Release of PPD from different electropsun scaffolds. (*b*) Model fitting with the release data based on different drug-loaded nanofibres.

As mentioned above, PPD has acquired enormous attention because of the predominant anti-cancer effect in experimental animals and cultured cells. But, the weak aqueous solubility and less bioavailability of PPD have limited its clinical application. There are a lot of drug dosage forms which are used to improve the bioavailability of PPD [10,33]. To the best of our knowledge, this is the first time that PPD-loaded nanofibres had been produced by eletrospunning. This kind of drug carrier can maintain the drug release for a long time and could be attached directly on the lesion site to reduce the system toxicity and increase the local drug concentration to improve the drug bioavailability preferably. The PPD release features were performed under stimulated physiological environment (buffer solution, pH 7.4) at 37°C for approximately 168 h time period. To research the drug release properties of drug-loaded nanofibres with different drug concentrations, the release profiles of PCL/3%PPD, PCL/5%PPD, PCL/10%PPD and PCL/20%PPD electrospun nanofibres containing the equivalent PPD contents were studied. Drugs could be distributed in two places at the nanofibres: the surface of nanofibres and inside the fibres. And there might be three different mechanisms of drug release: release of surface-loaded drug, diffusion and degradation of the carrier [11,34]. As shown in figure 7*a*, all specimens showed a rapid drug release rate in the first 12 h, especially for the beginning 6 h. The release of PPD from PCL/3%PPD, PCL/5%PPD, PCL/10%PPD and PCL/20%PPD nanofibres in 6 h was 15.6%, 18.52%, 20.68% and 23.08%, respectively. This result may be due to the release of surface-loaded PPD and part of the drugs which were close to the inner surface of fibres. To achieve enough initial dosage to kill tumours, the burst release of anti-neoplastic drugs is necessary. And for those that may survive from the initial burst, a continued release is also essential to prevent their further proliferation and migration [21]. In addition, we can see that the cumulative release of PPD from PCL/3%PPD, PCL/5%PPD, PCL/10%PPD and PCL/20%PPD nanofibres in 144 h was 29.53%, 34.06%, 38.51% and 50.07%, respectively, which was increased as the drug-loaded concentration increased. The sustained and slow PPD release allows the nanofibres with a coveted treated concentration of anti-cancer drug for a long time, which made them a potential carrier to kill cancer cells.

### 3.8. Release kinetics

The release kinetics of PPD is investigated by fitting several mathematical models to the release data. The Alfrey model has better agreement with the release data with higher *R*^2^ values for nanofibres with different concentrations of PPD as illustrated in figure 7*b*. The value of *R*^2^ of PCL/3%PPD, PCL/5%PPD, PCL/10%PPD and PCL/20%PPD nanofibres is 0.984, 0.980, 0.982 and 0.992, respectively. Two competing release mechanisms were used to explain this type of drug release kinetics: a Fickian diffusional release and a Case II transport release [35]. The molecular diffusion of the drug via chemical potential gradient is the cause of the Fickian diffusion model. A Case II transport release is associated with stresses and state transition in hydrophilic polymers which swell in water or biological fluids [36]. The drug release rate is independent of drug concentration in a Case II transport release. Meanwhile, in the Higuchi model, the fraction of drug released is proportional to the square root of time [37]. The release characteristics of PPD are consistent with previous reports.

### 3.9. *In vitro* anti-tumour effect

**Figure 8.**
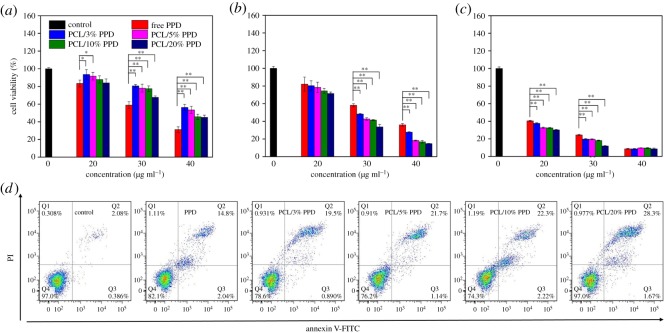
The viability of Hep-2 cells treated by PPD, PCL/3%PPD, PCL/5%PPD, PCL/10%PPD and PCL/20%PPD at an equivalent PPD dose of 20 µg ml^−1^, 30 µg ml^−1^ and 40 µg ml^−1^ for 1 (*a*), 3 (*b*) and 5 (*c*) days. (*d*) Annexin V/PI analysis of the apoptosis of Hep-2 cells treated with an equivalent PPD dose of 20 µg ml^−1^ after 3 days. The data are presented as average ± standard deviation (*n* = 5). Statistical significance: *p < 0.05; **p < 0.01.

**Figure 9.**
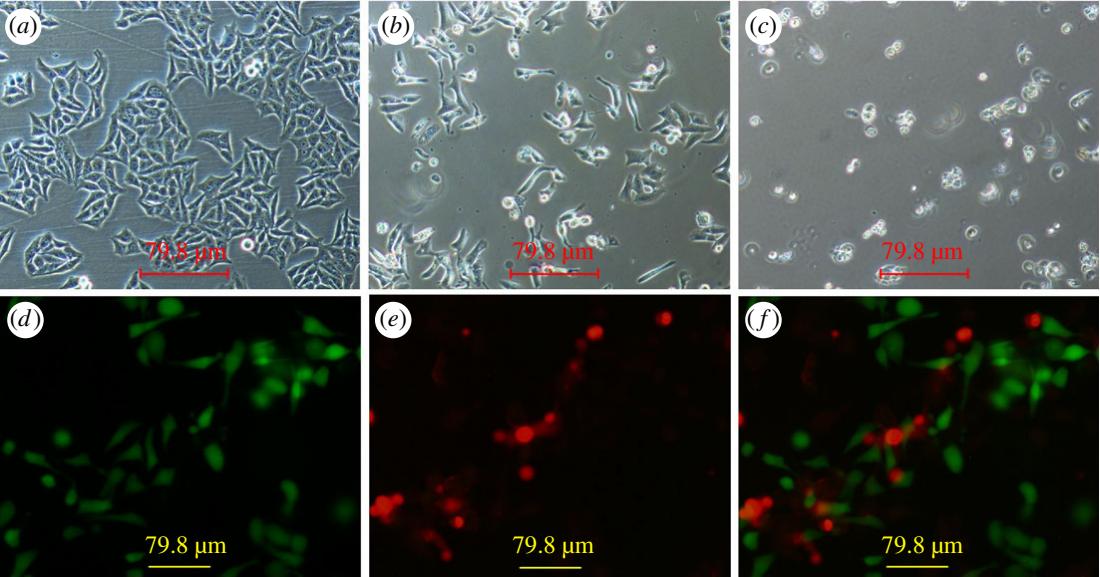
Microscopic images of Hep-2 cells. (*a*) Cells untreated with PCL/PPD nanofibres, (*b*) cells treated with PCL/PPD nanofibres for 72 h, (*c*) cells treated with PCL/PPD nanofibres for 120 h; (*d*–*f*) fluorescence images of Hep-2 cells. (*d*) Living cells, (*e*) dead cells and (*f*) merged images of (*d*) and (*e*).

It is significant for drug-containing materials to have a high ability of inhibiting cell proliferation. Thus, the *in vitro* anti-cancer activities of PPD-loaded nanofibres with different concentrations were examined in Hep-2 cancer cells by the MTT assay. Through preliminary experiments, we confirmed that PCL nanofibres alone were not effective in inhibiting the tumour cell growth. Figure 8*a*–*c* shows the cell viability of Hep-2 cells treated with different samples for 24 h, 72 h and 120 h incubation, respectively. As the drug concentration was increased, the cell viability was decreased. The experimental data show that both the pure PPD and PPD loaded in nanofibres suppresses the tumour cell proliferation in a concentration-dependent way, which is consistent with previous studies [7,38]. When the incubation time was 24 h, the free PPD group showed a better anti-tumour activity. When the incubation time increased to 72 h, drug-loaded nanofibre membranes gradually showed better anti-tumour effect than free PPD. It is proved that the cytotoxic effect of the tested specimen is gradually increased with increasing duration of the incubation period. This consequence is attributed to the sustained release of drug from the fibres. What is more, the anti-tumour efficacy of nanofibre membrane increased along with the increase of drug-loaded concentration. The reason for the phenomenon perhaps is the more drug release at the more drug-loaded nanofibres at the same condition. As shown in figure 8*d*, the percentage of apoptotic cells untreated and treated with free PPD, PCL/3%PPD, PCL/5%PPD, PCL/10%PPD and PCL/20%PPD was 2.6 ± 0.4, 16.8 ± 2.1, 20.3 ± 2.8, 22.8 ± 1.8, 24.5 ± 3.2 and 29.9 ± 2.4, respectively. The green and red fluorescence signals in figure 9*d*–*f* represent live and dead cells, respectively. Figure 9*a*–*c* intuitively shows the change in cell morphology under the treatment of the drug. These results indicate that these drug-loaded nanofibres we fabricated could be a potential candidate as a drug carrier for PPD.

### 3.10. *In vivo* anti-tumour effect

As shown in electronic supplementary material, figure S1, the mean tumour volume of the PPD group and the PCL/PPD nanofibre group is smaller than that of the control group and the PCL group. The mean tumour volume of the PPD group and the PCL/PPD nanofibre group is less than 140 mm^3^, while those of the other two groups are over 220 mm^3^. Although there was no significant difference in tumour volume between the drug-loaded nanofibre group and the drug group, one-time implantation of the nanofibre group displayed a comparable tumour growth suppression compared with the three-time injection of the PPD group after 9 days of treatment. This can be ascribed to several reasons. On the one hand, the drug-loaded nanofibre membrane could be implanted at the tumour site directly. The drug could be released near the site of the lesion in need of treatment, thereby reducing the drug loss, improving the bioavailability of the drug and effectively avoiding systemic side effects caused by systemic administration. On the other hand, the drug could be released from the drug-loaded nanofibres in a sustained way, ensuring therapeutic drug levels at the tumour site for an extended period. The drug release characteristics could reduce the number of administrations, avoid fluctuations in drug concentrations that occur during repeated administration, and reduce side effects and patient suffering. Therefore, the *in vivo* anti-tumour experiment demonstrated that the drug-loaded nanofibres evidently minimized administration frequency and drug dosage, which gives them a great advantage as a drug carrier.

## 4. Conclusion

We have successfully developed PPD-loaded PCL nanofibres to improve PPD bioavailability by electrospinning. The highly uniform, smooth and bead-free nanofibre was successfully fabricated. In this study, the results indicate that the incorporation of PPD changes the tensile strength of nanofibres. But, the addition of PPD has imperceptible influence on hydrophilicity and thermodynamic stability. All of the electrospun nanofibrous webs are equipped with perfect hydrophilicity and thermal stability. The drug-loaded nanofibres show a lasting anti-tumour effect. And the *in vitro* PPD release study manifested that the drug was released in a sustained drug release manner for a long period. These results indicate that the PCL nanofibre is an appropriate drug-delivery system for PPD, especially for the prevention of local tumour recurrence after surgery. As far as we know, this is the first report about utilization of electrostatic spinning nanofibre membrane loaded with PPD for the treatment of cancer. This research also provides an alternative method to enhance the clinical application of PPD.

## Supplementary Material

SEM Images of Nanofibers; Biodegradation Test of Nanofibers; Contact angle Measurement; FTIR Analysis; TGA analysis

## Supplementary Material

Ethical approval document

## Data accessibility

Our data are deposited at Dryad (http://dx.doi.org/10.5061/dryad.bt010) [39].
