# Supplementary material for: Polycaprolactone nanofibres loaded with 20(S)-protopanaxadiol for in vitro and in vivo anti-tumour activity study
Source: R Soc Open Sci. 2018 May 2;5(5):180137. doi: 10.1098/rsos.180137 (PMC5990777; doi:10.1098/rsos.180137)
Supplement: Ethical approval document [file rsos180137supp2.pdf]

# 吉林大学第二医院伦理委员会临床科学研究审批件

（2018 年）研审第（004）号

| | | | |
|---|---|---|---|
| 项目名称 | Polycaprolactone Nanofibers loaded with 20(S)-Protopanaxadiol for *In Vitro* and *Vivo* Antitumor Activity Study | | |
| 申请资助类别 | | | |
| 申请科室 | Department of Otolaryngology, Head and Neck Surgery | 项目负责人 | Bo Teng |
| 研究内容简介 | In this work, 20(S)-Protopanaxadiol (PPD) loaded polycaprolactone (PCL) nanofibers were successfully fabricated by electrospinning technique using tween-80 as a solubilizer. Firstly, smooth and continuous nanofibers were collected by using suitable solvent and appropriate spinning conditions. Secondly, nanofiber mats were characterized by scanning electron microscope (SEM), thermogravimetric (TG) analysis, Fourier transform infrared spectroscopy (FTIR) and mechanical test. Finally, nanofibrous membranes were evaluated using water contact angle, in vitro drug release, biodegradation test, in vitro and vivo antitumor activity and cell apoptosis assay. As a result, scanning electron microscopy observations indicated that diameter of the drug-loaded nanofibers increased with drug concentration increasing. Thermogravimetric (TG) analysis and mechanical test showed nanofibers equipped with great thermal and mechanical properties. Biodegradation test exhibited that the structure of fabricated nanofibers had a certain degree of change after 15 days. In vitro release study showed that PPD from drug-loaded nanofibers could be released in a sustained and prolonged mode. The cytotoxic effect of drug-loaded nanofiber mats examined on the human laryngeal carcinoma cells (Hep-2 cells) demonstrated that the prepared nanofibers had a remarkable antitumor effect. Meanwhile, the drug-loaded fiber mats showed a super antitumor effect in vivo antitumor study. All in all, PCL nanofibers could be a potential carrier of PPD for cancer treatment.<br>All experiments were performed in compliance with relevant national laws or guidelines; all experiments followed institutional guidelines, In accordance with the regulations of the Ministry of science and technology of the state on the guidance of good laboratory animals, and other laws and regulations, we agreed to carry out research. | | |

| | | | | |
|---|---|---|---|---|
| 伦理委员会审批意见 | ☑ 同意 | ☐ 作必要修改后同意 | ☐ 不同意 | ☐ 终止或暂停 |
| 伦理委员会主任委员签字 | [signature] | | | |

吉林大学第二医院伦理委员会

2018年 1 月3 日
